# Bitter taste receptors

**DOI:** 10.1093/chemse/bjaf064

**Published:** 2025-12-15

**Authors:** Maik Behrens, Silvia Schaefer

**Affiliations:** Leibniz Institute for Food Systems Biology at the Technical University of Munich, Freising, Germany; Leibniz Institute for Food Systems Biology at the Technical University of Munich, Freising, Germany; TUM Graduate School, TUM School of Life Sciences Weihenstephan, Technical University of Munich, Freising, Germany

**Keywords:** bitter taste receptors, review, taste perception, gustatory system, extraoral

## Abstract

Hundreds of bitter substances, synthetic or natural, toxic or health beneficial, chemically complex organic molecules or simple inorganic metal salts, surround us and other vertebrates. Their detection is mediated by bitter taste receptors present in the oral cavity and beyond. The present review article summarizes the current knowledge about these highly versatile receptors in humans and other vertebrates. Following the introductory description of taste anatomy and canonical taste signal transduction, a brief section about bitter compounds provides a flavor of their chemical diversity. The main part of the article is devoted to the human bitter taste receptors, their agonist profiles, structures, and sensitivities. For comparison, a section of bitter taste receptors in other species is added, and, to highlight the functional complexity of these molecules, nongustatory bitter taste receptors and their functions are described.

## Introduction

The sense of taste in vertebrates is important to guide food intake toward nutritionally valuable food items and away from non-nutritious or even harmful sources (for a recent review, see Behrens 2025). The required assessment of food before consumption relies on the rapid analysis of chemical stimuli representative of the five basic taste qualities, sweet, sour, salty, umami, and bitter, by taste-receptive proteins in the oral cavity (Lindemann 1996). These taste receptors are expressed in sensory cells, which occur in congregates of about 100 cells, the taste buds (Chaudhari and Roper 2010). During processing, the chemical signals are transformed into electrical impulses and transmitted to the brain, where they induce adequate consummatory behaviors. For most animals as well as humans, sweet and umami tastes are appetitive, whereas sour and bitter tastes are aversive. Depending on the concentration, salty perception may serve as a promoter of ingestion at low concentrations or an aversive signal at high concentrations (Lindemann 1996). Although for many years two hypotheses of taste signal processing were heavily disputed (Smith et al. 2000), namely the “across-fiber pattern theory,” which suggested that the collected peripheral taste signals are to some degree mixed with regard to the five basic taste qualities, and the “labeled-line theory” that favored a separate transmission of taste quality information from the taste receptor cell to the brain, nowadays the dispute is more or less settled. The “labeled-line theory” is broadly accepted at least as the dominant signal processing pathway by most researchers. In fact, taste signaling starts from taste receptor cells that are devoted to detect only one of the five basic taste qualities (Yarmolinsky et al. 2009). These highly segregated types of taste-receptive cells are characterized by nonoverlapping expression of the different taste receptors in separate cell populations. Due to the fact that multiple receptors for the bitter taste quality exist in most vertebrates, the bitter taste receptor cell population is separated into sub-populations, each expressing only a subset of bitter taste receptor genes (Behrens et al. 2007). Despite their initial naming as “taste receptors” (Adler et al. 2000; Chandrashekar et al. 2000; Matsunami et al. 2000), it turned out that these receptors occur in numerous tissues outside the oral cavity and that their functions are not limited to gustation (for comprehensive recent reviews, see Wang et al. 2020; D'Urso and Drago 2021; Tuzim and Korolczuk 2021; Behrens and Lang 2022). This article summarizes current knowledge on bitter taste receptors, a highly complex family of G protein-coupled receptors.

## Taste anatomy

### Peripheral

**Fig. 1.**
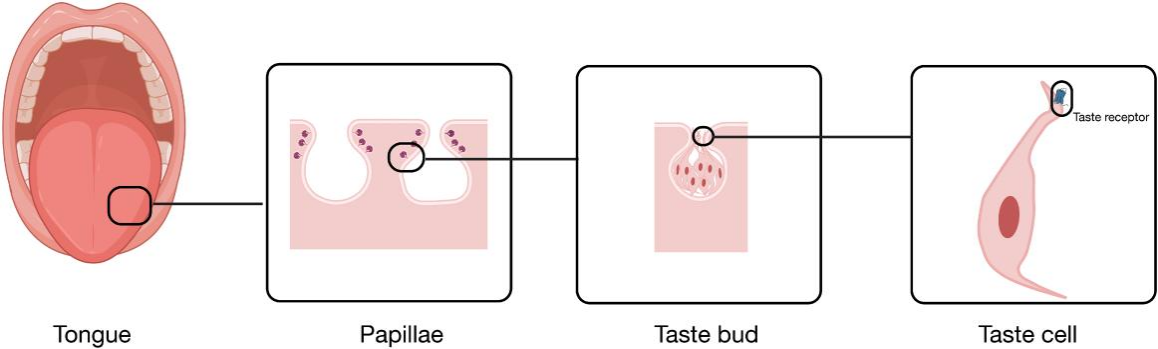
Schematic representation of the peripheral taste anatomy. The tongue surface contains papillae with embedded taste buds. Each taste bud comprises multiple taste receptor cells that detect chemical compounds. Signals are transmitted via afferent nerve fibers to the brain, where taste information is processed. Created in BioRender. Schäfer, S. (2025) https://BioRender.com/pwr9umi.

In humans and most vertebrates, the structures responsible for the detection of gustatory signals are called taste buds (Fig. 1). On the human tongue, taste buds are located within three types of gustatory papillae, named foliate (on both sides of the posterior tongue), circumvallate (on the posterior top of the tongue), and fungiform papillae (on the apical tongue surface) (Witt and Reutter 2015). Altogether, around 5,000 taste buds can be found in the oral cavity, the larynx, and pharynx, as well as the epiglottis (Witt and Reutter 2015). The taste buds themselves merge ∼100 cells organized in an onion-like shape (Yang et al. 2020). The taste bud cells fall into different types with distinct functions. The most abundant are Type I cells, which comprise approximately half of the total number of taste cells (Roper and Chaudhari 2017). Interestingly, Type I cells express a glutamate transporter (GLAST), suggesting an involvement in glutamate uptake, which is considered a possible neurotransmitter in taste buds (Lawton et al. 2000). The best established taste neurotransmitter is adenosine triphosphate (ATP) (Finger et al. 2005), for which a plasma membrane-bound nucleotidase (NTPDase2) can be found in Type I taste cells to govern extracellular hydrolyzation (Bartel et al. 2006). Furthermore, a role in the elimination of potassium (K^+^) cations in the interstitial spaces of the taste buds was proposed, causing a decreased chance of other taste cell types to become activated (Chaudhari and Roper 2010). Recently, Rodriguez et al. found that Type I cells actively contribute to the modulation of Type II cell signaling (Rodriguez et al. 2021). They respond to the neurotransmitter ATP that is released by activated Type II cells and, in turn, may signal back to Type II cells via GABAergic input. It was suggested that this intricate association of the two cell types resembles the tripartite synapses formed between neurons and astrocytes in the central nervous system. These results were confirmed and expanded by a study by Park and colleagues, who demonstrated the involvement of Type I cells in sweet taste adaptation (Park et al. 2025). Using genetically modified P2RY2 knockout mice, it was shown that ATP released from sweet-responsive Type II cells not only stimulated P2X_2/3_ purinergic receptors located on afferent nerve fibers to transmit taste information to the brain (Finger et al. 2005) but also elicited responses in Type I cells. These responses were mediated through P2RY2 purinergic receptors and triggered delayed and prolonged responses in Type I cells, causing inhibitory feedback on sweet taste transmission. All this suggests a role in ion homeostasis as well as in the modulation and termination of signals, arguing that Type I cells represent glia-like cells. Another novel feature of Type I cells has been reported by Wilson and colleagues, who observed that Type I cells engulf apoptotic Type II and Type III cells (Wilson et al. 2025). Hence, Type I cells may phagocytose dying cells and could therefore be involved in the clearing of taste buds.

While Type I cells were initially considered potential candidates for mediating amiloride-sensitive salt responses (Vandenbeuch et al. 2008), their exact contribution remains unclear. Although amiloride-sensitive currents have been detected in these cells, current findings do not support a prominent role of Type I cells in salt transduction. Instead, studies have shown that ENaC-expressing taste cells co-express CALHM channels and release ATP as a neurotransmitter, indicating that amiloride-sensitive salt taste may primarily be mediated by Type II–like cells rather than by Type I cells (Nomura et al. 2020).

Type II cells, also often referred to as taste receptor cells, contain the TAS1R as well as the TAS2R G protein-coupled receptors (Chandrashekar et al. 2006). Further, Type II cells also express channels crucial for the generation of action potentials, such as voltage-gated potassium and sodium channels (Gao et al. 2009; Wang et al. 2009). With few exceptions (Yoshida and Ninomiya 2016), Type II cells express G protein-coupled receptors responsive to either sweet (TAS1R2 + TAS1R3), umami (TAS1R1 + TAS1R3), or bitter (TAS2R2) taste in a strictly segregated fashion. Even though these taste cells play a central role in taste perception and taste signal transduction, they do not express typical synaptic vesicles. Recent studies have shown that calcium homeostasis modulators 1 and 3 (CALHM1 and CALHM3) are selectively enriched in Type II taste receptor cells, where they assemble into a functional channel complex (Taruno et al. 2021). Genetic ablation of either subunit eliminates taste responses to sweet, bitter, and umami stimuli, highlighting the essential role of the CALHM1/3 complex as the primary adenosine triphosphate (ATP) release mechanism downstream of GPCR-mediated taste transduction (Taruno et al. 2013; Ma et al. 2018). Intriguingly, the ATP involved in signal transmission of Type II cells (Finger et al. 2005) is produced by atypical mitochondria, which are found in close contact to CALHM subunits and purinergic nerve fibers (Romanov et al. 2018).

In contrast to that, Type III cells harbor conventional synapses, which are characterized by the presence of synapse-specific proteins, such as SNAP25 (25 kDa synaptosomal-associated protein), a SNARE protein mediating the release of transmitter substances from synaptic vesicles (Antonucci et al. 2016), as well as other components such as voltage-gated calcium channels, biogenic amine and GABA neurotransmitters (DeFazio et al. 2006). Using epithelial cell-specific SNAP25 knockout mice, it has been shown that, unlike Type II cells, information transmission from Type III cells involves vesicular synaptic transmission (Horie et al. 2025).

Moreover, the Type III cells are also involved in sour taste transduction (Huang et al. 2008). It turned out that two components contribute to sour taste reception: (i) the proton channel otopetrin-1 (Otop-1), which allows the apical entry of protons (Teng et al. 2019; Zhang et al. 2019), and (ii) the intracellular acidification and subsequent blockage of potassium channels caused by the diffusion of weak organic acids through the plasma membrane followed by dissociation (Lyall et al. 2001; Richter et al. 2003).

Recent research also postulates the existence of a broadly tuned subset of Type III cells using a PLCβ3 signaling pathway to respond to umami, sweet, and bitter stimuli (Banik et al. 2020). Another kind of cell found in taste buds is the Type IV cells. These are postmitotic and function as precursors for all other mature taste bud cells (Chaudhari and Roper 2010; Barlow 2022). Their shape is described as ovoid, and they are located at the base of the taste bud; thus, their alternative name is basal cells (Yang et al. 2020).

**Fig. 2.**
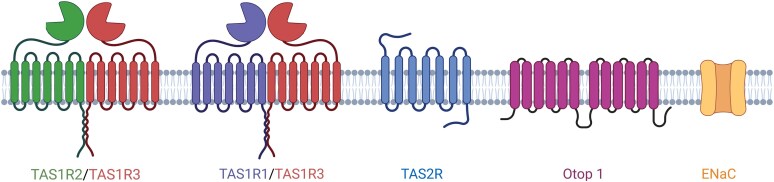
Molecular basis of taste perception. Sweet taste is mediated by the TAS1R2/TAS1R3 heterodimer, while umami is detected by the TAS1R1/TAS1R3 heterodimer. Bitter compounds are sensed by ∼25 TAS2R receptors. Sour taste perception involves the proton channel Otop1, and salty taste is mediated by the epithelial sodium channels (ENaC), of which the exact subunit composition requires confirmation. Created in BioRender. Schäfer, S. (2025) https://BioRender.com/uyllcla.

Bitter, umami, and sweet tastants activate different GPCRs (Fig. 2). GPCRs are built from seven transmembrane domains, connected by three extracellular (ECL) and three intracellular loops (ICL). In general, taste receptors are divided into two families, called the taste 1 receptor family (TAS1R) and the taste 2 receptor family (TAS2R) (Chandrashekar et al. 2006). Umami and sweet taste receptors belong to the TAS1R gene family, which consists of the three members TAS1R1, TAS1R2, and TAS1R3. For functional receptors, TAS1R2 and TAS1R1 heterodimerize with TAS1R3 as the common subunit (Hoon et al. 1999; Nelson et al. 2001, 2002; Li et al. 2002). The receptors of the TAS1R family belong to the class C of GPCRs (Hoon et al. 1999; Li et al. 2002). This receptor class is characterized by a large extracellular amino-terminal domain, containing a Venus flytrap module (VFM) and a cysteine-rich domain (CRD), which connects the VFM with the transmembrane domains (Chun et al. 2012; Roper and Chaudhari 2017). The heterodimer combination of TAS1R2/TAS1R3 reacts to various sugars, some D-amino acids, and synthetic and natural high-intensity sweeteners (Lindemann 2001; Nelson et al. 2001). The subunits have various binding sites; thus, the extracellular domain of either TAS1R2 or TAS1R3 binds numerous sugars and sugar alcohols (Nie et al. 2005). In the cleft of the VFM, low-molecular-weight sweet compounds such as aspartame and others can bind (Liu et al. 2011; Masuda et al. 2012; Maillet et al. 2015). The recognition of the sweet proteins, such as brazzein and thaumatin, depends on the CRD (Jiang et al. 2004; Masuda et al. 2013); the binding of some sweeteners, for instance cyclamate or neohesperidin dihydrochalcone (Winnig et al. 2007), occurs on residues of the transmembrane domain of TAS1R3 (Jiang et al. 2005b), which has also been proven as binding site for the selective inhibitor of the human sweet taste receptor, lactisole (Winnig et al. 2005; Jiang et al. 2005a). On the contrary, cells expressing the heterodimer TAS1R1/TAS2R3 are dedicated to umami taste (Li et al. 2002; Nelson et al. 2002). They detect L-amino acids, particularly glutamate and aspartate, in humans (Ikeda 2002). A widely known agonist, eliciting umami taste, is monosodium glutamate (MSG). A synergistic increase of taste intensity is achieved when glutamate is combined with low quantities of 5′ ribonucleotides, such as inosine monophosphate (IMP) (Nelson et al. 2002; Roper and Chaudhari 2017).

### Central

**Fig. 3.**
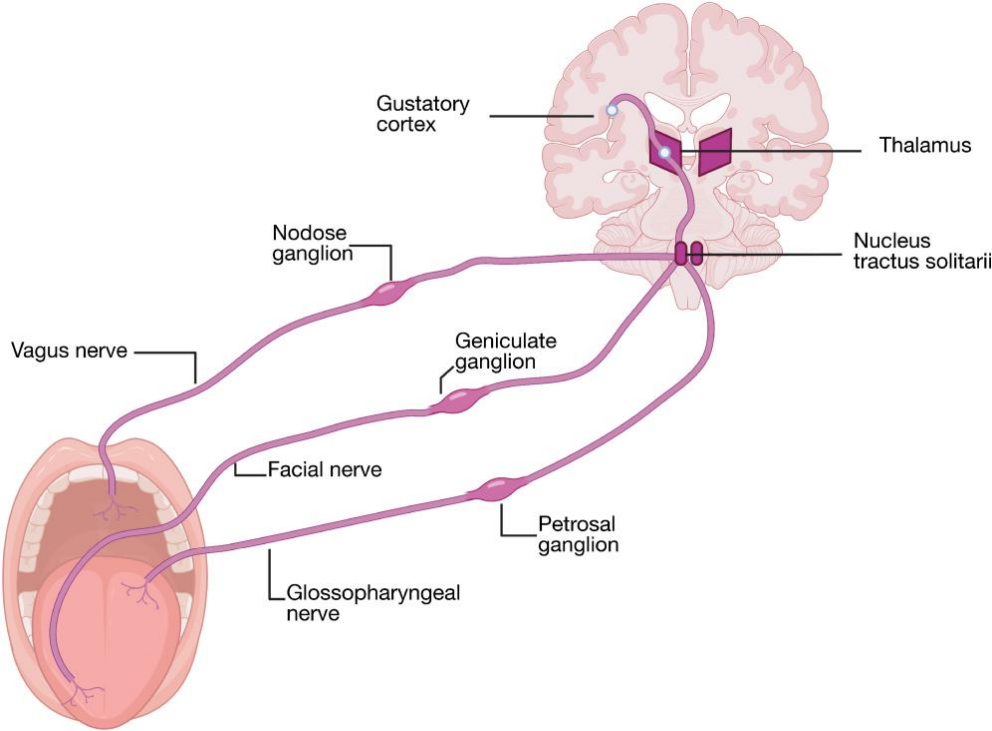
Neural pathways of taste perception. Taste information from the tongue is transmitted via the facial (cranial nerve VII, cell bodies in geniculate ganglion), glossopharyngeal (cranial nerve IX, petrosal ganglion), and vagus (cranial nerve X, nodose ganglion) nerves to the nucleus of the solitary tract in the brainstem. Signals are then relayed to the thalamus and subsequently to the gustatory cortex, where taste perception is processed further. Created in BioRender. Schäfer, S. (2025) https://BioRender.com/570qkwl.

The gustatory pathway can be traced from the tongue toward central processing in the brain (Fig. 3). Taste information transmission is mediated by three cranial nerves: the facial nerve (CN VII), which innervates the anterior two-thirds of the tongue via the chorda tympani nerve branch and contributes fibers to the soft palate through the greater petrosal branch; the glossopharyngeal nerve (CN IX), which carries information from the posterior third of the tongue; and the vagus nerve (CN X), which conveys taste transmission from the throat and epiglottis. All taste fibers are classified as special visceral afferents (Myckatyn and Mackinnon 2004). The primary sensory cell bodies associated with these nerves are localized in the geniculate ganglion (CN VII), the inferior or petrosal ganglion (CN IX), and the nodose ganglion (CN X). Their central projections enter the brainstem and converge in the rostral nucleus of the solitary tract (nucleus tractus solitarius), the primary relay station for gustatory input. While the rostral portion of this nucleus processes taste, the caudal region integrates visceral and cardiopulmonary signals (de Castro and Marrone 2025). From the gustatory nucleus, second-order neurons project ipsilaterally to the parvocellular division of the ventral posteromedial nucleus of the thalamus (VPM pc). Subsequent third-order fibers ascend via the internal capsule to terminate in the frontal operculum, anterior insula, and rostral segment of Brodmann area 3B, regions collectively responsible for fine discrimination of taste qualities (Gibbons and Sadiq 2025). Finally, descending and associative projections link the gustatory cortex to the orbitofrontal cortex (OFC), a hub for the integration of taste and olfactory cues. At this level, gustatory signals contribute to higher-order processes such as flavor perception and the rewarding aspects of food consumption (Small et al. 2007; Rolls 2023).

## Taste signal transduction

**Fig. 4.**
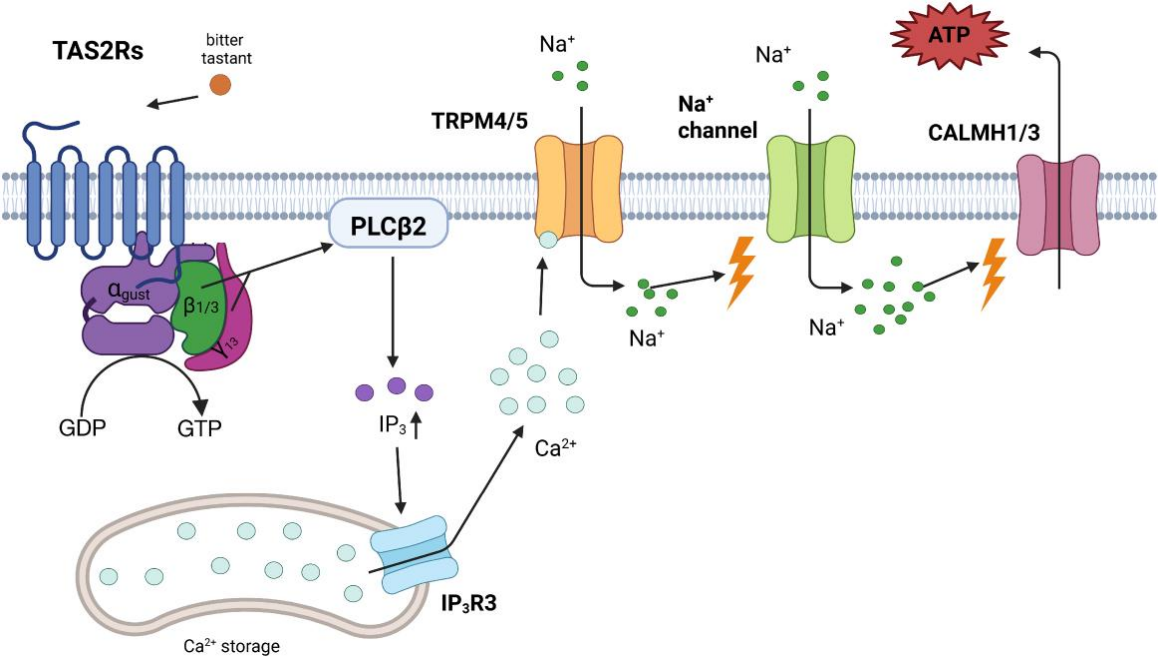
Mechanism of taste GPCR signal transmission exemplified for bitter taste. In Type II cells, bitter, sweet, or umami tastants bind to the GPCR and activate a canonical taste-specific phosphoinositide pathway, increasing intracellular Ca2^+^ levels. This depolarises the membrane via the cation channel TRPM4/5. The converged action of increased intracellular Ca^2+^ and membrane depolarisation opens large voltage-gated channels, leading to ATP release. Shown here is a TAS2R monomer (bitter taste transduction) with a small extracellular domain. TAS1R taste GPCRs would be heterodimers with larger extracellular domains. Modified from Chaudhari and Roper (2010). GPCR: G protein-coupled receptor; Ca^2+^: calcium ions; Na^+^: sodium ions; TRPM4/5: transient receptor potential cation channel subfamily M members 4 and 5; TAS2R: taste 2 receptor member; CALMH1 and 3: calcium homeostasis modulator 1 and 3; IP_3_R3: 1,4,5-trisphosphate receptor type 3; IP_3_: inositol 1,4,5-trisphosphate. Created in BioRender. Schäfer, S. (2025) https://BioRender.com/t18wpvu.

In the oral cavity, TAS2Rs are located on the surface of taste receptor cells so they can interact directly with possible tastants (Behrens et al. 2012). The activation of bitter taste receptors starts a signaling cascade (Fig. 4). Upon ligand binding, the TAS2R's conformation changes, and the heterotrimeric G protein dissociates into G_α_-gustducin (McLaughlin et al. 1992) and the G_βγ_ dimer (Rössler et al. 2000). The latter interacts with phospholipase Cβ2 (PLCβ2) (Huang et al. 1999; Zhang et al. 2003). PLCβ2 mediates the synthesis of inositol 1,4,5-trisphosphate (IP_3_), an intracellular second messenger (Huang et al. 1999). The increase of IP_3_ leads to the activation of inositol 1,4,5-trisphosphate receptor type 3 (IP_3_R3) (Clapp et al. 2001), which results in releasing Ca^2+^ from the endoplasmic reticulum (ER) into the cytosol (Ogura et al. 2002). The increased intracellular Ca^2+^ levels cause an opening of transient receptor potential cation channel subfamily M members 4 and 5 (TRPM4/5) (Pérez et al. 2002; Banik et al. 2018), leading to a depolarization by the influx of Na^+^ cations. If large enough, action potentials are evoked in receptor cells (Yoshida et al. 2018) and trigger the activation of voltage-gated ion channels, namely calcium homeostasis modulator 1 and 3 (CALMH1/3) (Taruno et al. 2013; Ma et al. 2018), through whose pores the taste bud neurotransmitter ATP (Finger et al. 2005) is released (Taruno et al. 2013; Ma et al. 2018).

## Bitter compounds

Among the primary taste stimuli, the group of bitter substances is most diverse, and the number of identified activators exceeds 1,000 by far (Table 1). The BitterDB database (Ziaikin et al. 2025) contains more than 2,000 compounds of which ∼700 have been associated with at least one vertebrate bitter taste receptor. A large fraction of the bitter compound-receptor interactions that are listed in BitterDB originate from functional heterologous expression experiments with human TAS2Rs (e.g. Meyerhof et al. 2010). Despite the enormous number of cognate bitter compounds, very few features that qualify chemicals to taste bitter/activate at least one of the human TAS2Rs have been identified. One exception is the isothiocyanate/thiourea group of compounds, which specifically activate the human TAS2R38 (Bufe et al. 2005). This group consists of numerous important synthetic and natural bitter substances. Another exception is the bitter glycosides that specifically activate human TAS2R16, if the bound sugar moiety is glucose or some other hexoses in β-D-conformation (Bufe et al. 2002). A third group may just emerge in the form of bitter-tasting peptides. While bitter peptides represent a highly complex agonist group, most of them target a subset of 5 TAS2Rs, namely TAS2R1, -R4, -R14, -R39, and -R46 (Kohl et al. 2013). In case of peptides, the sheer number of potentially bitter peptides, e.g. a simple trimeric peptide built from the 20 biogenic amino acids, allows for 20^3^ (=8,000) permutations with an enormous steric flexibility, making it very difficult to narrow down the actual pharmacophores that activate TAS2Rs. However, it is very likely that the binding pockets of TAS2Rs will not accommodate peptides exceeding a maximal size, and hence, large peptides could be reduced to smaller bitter epitopes. Moreover, it is evident that some amino acids occur in bitter peptides with strongly elevated or reduced frequencies (Steuer et al. 2025). This, together with additional parameters such as hydrophobicity scores (Steuer et al. 2025), indicates that rather limited bitter peptide pharmacophores might exist. Good examples for this are provided by previous observations. Already in one of the early reports on bitter peptides by Kohl and colleagues (Kohl et al. 2013), it was found that the TAS2R39 responds rather to the indole side-chain of tryptophane than to L-tryptophane (as well as D-tryptophane in this case) itself, and hence the receptor should interact with all peptides containing tryptophane, if it is sufficiently exposed to the receptor's ligand binding site. Another example is arising from cyclic peptides from linseed oil, the so-called cyclolinopeptides (CL), which activate two TAS2Rs, but only when they contain methionine residues that were previously oxidized to methionine sulfoxide or methionine sulfone groups (Lang et al. 2022). This indicates that neither the amino acid sequences nor the cyclic nature of the CLs contribute to their bitterness per se; they could merely serve as “presenters” of the critical oxidized methionine. Altogether, the number and complexity of bitter peptides make this type of bitter agonist a perfect test case for computational approaches, which have been undertaken in recent years (Charoenkwan et al. 2020; Zhang et al. 2023; Kuhfeld et al. 2024; Srivastava et al. 2024; Yu et al. 2024). The most recent addition to these attempts to make the bitterness of peptides better predictable has applied machine learning algorithms to a large collection of approximately 1,000 experimentally verified bitter and nonbitter peptides (Steuer et al. 2025). This dataset has been made publicly available (called Bitter Peptide Space (BPS-) 1,000; https://bps1000.leibniz-lsb@tum.de/). Briefly, the outcome of this approach suggested that, (i) the amino acids F, G, I, L, P, R, V, W, and Y occur most frequently in bitter peptides, whereas A, D, E, M, S, and T are more frequently found in nonbitter peptides; (ii) peptides with Q-values (=an estimation of a peptide's hydrophobicity; Tanford 1962) above 1,400 tend to be bitter, whereas peptides with Q-values below 1,300 are less likely to exert bitterness; (iii) according to another value indicating hydrophobicity, the logP-value, a logP-value above −0.20 is predictive for bitter peptides, however, a strict correlation was not evident. The study also revealed that bitter peptides are rather agonists of moderate to low potency, since the bitter taste thresholds for the curated dataset ranged between low micromolar and high millimolar concentrations. Future studies will certainly narrow down the molecular features of bitter peptides even further, and then it remains to be seen how large the apparent complexity of bitter peptides may in fact be. However, the vast majority of bitter compounds are chemically diverse and devoid of currently recognizable pharmacophores (Table 1).

**Table 1.** List of select chemically diverse bitter substances of natural and synthetic origin.

Origin	Chemical class	Compound
**Synthetic**	Acetamide	Acetaminophen (antipyretic, analgesic)
	Purine analog	Azathioprine (immunosuppressant)
	Aminoquinoline	Chloroquine (antibiotic)
	Aminoalkyl ether	Diphenhydramine (antihistamine)
	Alkyl-phenylketone	Haloperidol (neuroleptic)
	Imidazolethione	Methimazole (antithyroid)
	Diphenylmethane	Diphenidole (antiemetic)
	Morphinan	Dextromethorphan (antitussive)
	Anisole	Guaifenesin (expectorant)
	Naphthalene	Naphazoline (decongestant)
	Carbamate ester	Carisoprodol (muscle relaxant)
	Benzothiazole	Saccharin (sweetener)
	Chlorobenzene	Chlorhexidine (antiseptic)
	Benzoic acid	Benzoic acid (food preservative)
**Natural**	Cinchona alkaloid	Quinine (antimalarial)
	Indole alkaloid	Strychnine
	Glycoside	Salicin (analgesic, antiinflammatory)
	Xanthine	Caffeine (stimulant)
	Triterpene lactone	Absinthin
	Germancranolide	Parthenolide
	Inorganic	Magnesium sulfate (laxative, anticonvulsant)
	γ-butyrolactone	Andrographolide
	Secoiridoid glycoside	Amarogentin
	Lanostane	Cucurbitacin E
	Fatty alcohol	Falcarindol
	Iso-α-acid	Humulone
	Isoquinoline alkaloid	Noscapine (antitussive)
	picrotoxane sesquiterpenoid	Picrotoxinin

## Human bitter taste receptors

### Discovery and nomenclature

Human bitter taste receptors have been discovered, together with their rodent counterparts, more than 20 years ago by independent research groups (Adler et al. 2000; Chandrashekar et al. 2000; Matsunami et al. 2000). As the bitter taste receptors were found subsequently to the sweet and umami genes coding for the taste quality-specific subunits (the TAS1R3 subunit was discovered only later) (Hoon et al. 1999), then coined taste 1 receptors, the bitter taste receptors were named taste 2 receptors (Adler et al. 2000). In the following years, additional researchers entered the field, leading to parallel nomenclatures, which resulted in difficulties in clearly identifying these receptors in publications with universally applicable gene symbols. Unfortunately, a rather historically influenced and somewhat innovative use of nomenclature is still found in some publications, although meanwhile the gene symbols have been officially defined by the Human Genome Organization (HUGO) Gene Nomenclature Committee (https://www.genenames.org/tools/search/#!/?query=TAS2R). For human bitter taste receptors, the official gene symbols are TAS2R plus a one to two-digit number for gene products, and for genes, the symbols must be italicized (*TAS2R*). We will stick to this nomenclature throughout.

### The TAS2R gene family

It has been noticed right away that TAS2Rs represent typical GPCRs consisting of seven transmembrane domains that are connected via three intra- and three extracellular loops (Adler et al. 2000; Chandrashekar et al. 2000; Matsunami et al. 2000). The short amino terminus is located on the extracellular side, and the carboxyl terminus is located intracellular. Despite this typical topography, the integration of TAS2Rs into the superfamily of GPCRs turned out to be difficult because of the very limited homology with other existing GPCRs. Whereas some researchers favored categorizing the TAS2R gene family separately from all other GPCRs (Horn et al. 2003), others grouped them loosely with the frizzled receptors into a frizzled/TAS2 family (Fredriksson et al. 2003). More recently, phylogenetic analyses underscored the relationship of *TAS2R* genes with the large rhodopsin family of GPCRs (Nordström et al. 2011). Another obstacle has been the assessment of the size of the *TAS2R* gene family. Based on a mixture of an incomplete human genome project, the enormous variability of TAS2R genes in the human population (Kim et al. 2005), and hence, the differential assignment of segregating pseudogenes, the number of putative functional TAS2Rs fluctuated for many years and still does. The number of about 25 existing putatively functional TAS2R genes is mostly found in the literature and justified, considering the high individual variation found in human genomes. While some TAS2Rs, such as TAS2R9 (Dotson et al. 2008) and TAS2R38 (Kim et al. 2003; Bufe et al. 2005), exist in well-recognized functional and nonfunctional variants, additional variability of TAS2R46 (Kim et al. 2005; Roudnitzky et al. 2015) (early-stop mutation), TAS2R45 and TAS2R43 (Pronin et al. 2007; Roudnitzky et al. 2011, 2016) (copy-number polymorphisms/gene deletions) exists. The most recent finding that the firmly annotated pseudogene TAS2R2 (despite the report by Go et al., who recognized a potentially functional variant of this receptor already in 2005; Go et al. 2005) exists in some populations as a 26th functional TAS2R (Risso et al. 2017; Lang et al. 2023), emphasizes nicely why it is so difficult to call the exact size of the human TAS2R gene repertoire.

Already the first report on the functional characterization of mammalian bitter taste receptors (Chandrashekar et al. 2000) revealed that this group of chemosensory receptors shares a common problem with other chemosensory GPCR families. It was noticed that functional expression of TAS2Rs in heterologous cells required amino-terminal extensions (also called “export-tags”), thus paralleling previous observations for heterologously expressed odorant (OR) (McClintock et al. 1997) and pheromone receptors (V2Rs) (Loconto et al. 2003). The two mainly used export-epitopes for functional expression of bitter taste receptors are the first 39 amino acids of bovine rhodopsin (rho-tag) (Chandrashekar et al. 2000) and the first 45 amino acids of rat somatostatin receptor 3 (sst3-tag) (Bufe et al. 2002), both deriving from GPCRs exhibiting efficient cell surface translocation in heterologous cells. Later, it became evident that not all human TAS2Rs are strictly dependent on one of the amino-terminal extensions, indicating that bitter taste receptors have individual requirements for their cellular environment (Behrens et al. 2006). The latter is further underscored by the discovery of auxiliary factors aiding in the functional expression of some, but not all TAS2Rs (Behrens et al. 2006). The finding that TAS2Rs are glycoproteins and that their function depends on asparagine-linked glycan-structures located in the center of their 2nd extracellular loops indicates a complex biosynthetic pathway (Reichling et al. 2008).

### The agonist profiles of TAS2Rs

As already mentioned, in its recent version, the most comprehensive online collection of bitter compounds and bitter taste receptors, the BitterDB, holds ∼2,400 bitter compounds, and for ∼800 of these compounds an interaction with one or more TAS2Rs is shown (Ziaikin et al. 2025). Therefore, on average, each of the 26 human TAS2Rs should interact with ∼31 to ∼92 bitter compounds. For the latter number, it is assumed that every bitter-tasting compound needs to activate at least one of the functional TAS2Rs. Table 2 contains a list of all TAS2Rs and the number of agonists associated with them (status of July 9th, 2025). Clearly, some TAS2Rs exceed the average number of ∼31 by far, whereas others interact with fewer agonists. Hence, the average tuning breadths deviate considerably, with TAS2R1, -R4, -R5, -R7, -R10, -R14, -R38, -R39, -R43, and -R46 detecting an above average number of bitter compounds. It needs to be mentioned, however, that the data shown in Table 2 are certainly biased by a variety of factors. On the one hand, some TAS2Rs received far more attention than others, leading to more screening campaigns and thus, more identified bitter substances. This is particularly true e.g. for the TAS2R14, because of its extraordinary tuning breadth and as a potential drug target (e.g. Behrens et al. 2004; Roland et al. 2011; Levit et al. 2014), the TAS2R38, as the prime example for functional variability (e.g. Bufe et al. 2005; Wooding et al. 2010; Behrens et al. 2013; Verbeurgt et al. 2017), and the TAS2R16, which appears to be functionally highly conserved in many species (e.g. Bufe et al. 2002; Sakurai et al. 2010a; Imai et al. 2012; Thomas et al. 2017). On the other hand, bitter compounds/compound groups have received special interest, again leading to a certain bias in the functional characterization of select TAS2Rs. Among the numerous examples for bias introduced by preferential screening of compounds are TAS2R14 (drugs, drug-like compounds; Levit et al. 2014; Karaman et al. 2016; Thawabteh et al. 2019), TAS2R14 and TAS2R39 (flavonoids; Roland et al. 2011, 2013), as well as TAS2R16 (Bufe et al. 2002; Sakurai et al. 2010b; Thomas et al. 2017) and TAS2R38 (Bufe et al. 2005; Wooding et al. 2010; Behrens et al. 2013) because of their compound class selectivity. Therefore, it is important to compare the numbers of identified agonists with less biased screening campaigns. The numbers of agonists identified in those screening campaigns are provided in Table 2, next to the BitterDB row. It is evident that there is a good correlation between the BitterDB agonist numbers and the numbers obtained by screening campaigns with reduced bias. Some discrepancies, however, are evident, including TAS2R5, -R7, and -R20. In case of TAS2R5 specific screening of this receptor with derivatives of 1,10-phenanthroline, one of the originally identified agonists (Meyerhof et al. 2010), resulted in the identification of numerous additional phenanthrolines as TAS2R5 agonists (Kim et al. 2020).

**Table 2.** Number of bitter compounds identified for each TAS2R.

TAS2R	Number of bitter compounds in BitterDB	Sum of agonists after balanced screening
**1**	82	18
**2**	8	8
**3**	2	1
**4**	59	17
**5**	35	4
**7**	34	8
**8**	18	4
**9**	3	0
**10**	54	44
**13**	7	2
**14**	385	52
**16**	28	8
**19**	0	0
**20**	20	2
**30**	22	10
**31**	14	8
**38**	37	22
**39**	122	16
**40**	19	8
**41**	10	2
**42**	1	0
**43**	41	19
**45**	0	0
**46**	113	42
**50**	4	2
**60**	0	0

The second row displays the number of agonists listed in BitterDB. The third row displays the sum of the identified compounds by screenings with rather balanced bitter compound libraries (Meyerhof et al. 2010; Thalmann et al. 2013; Lossow et al. 2016; Lang et al. 2023).

### TAS2R-antagonists

The fact that a bitter taste is considered unpleasant by most individuals has raised strong interest in molecules or processes able to mask this sensation. Before functional heterologous expression assays achieved the deorphanization of numerous TAS2Rs, which has been a prerequisite for the subsequent discovery of molecules able to suppress the activation of specific TAS2Rs, several strategies to identify bitter masking agents were employed successfully and summarized in a comprehensive review article by Ley (2008). This brief section will focus on those bitter receptor antagonists that were discovered later through functional experiments, and hence, rather receptor-specific antagonists. The first receptor-specific antagonist discovered was 4-(2,2,3-trimethylcyclopentyl)butanoic acid, also known as GIV3727 (Slack et al. 2010). The synthetic compound was identified in a screening for molecules able to suppress the bitter off-taste of saccharin and acesulfame K via TAS2R31 and TAS2R43. Indeed, both receptors could be completely blocked with IC_50_-concentrations of GIV3727 in the low micromolar range (Slack et al. 2010). Soon thereafter, the first natural bitter taste receptor antagonists were discovered. It was demonstrated that 3β-hydroxydihydrocostunolide (3HDC) and 3β-hydroxypelenolide (3HP) showed antagonism against the receptor TAS2R46 (Brockhoff et al. 2011). Interestingly, these natural bitter antagonists occur together with powerful TAS2R46 activators, such as absinthin (Brockhoff et al. 2007), in wormwood. More detailed analyses revealed that 3HP could not repress TAS2R46 activation by more than 50%, and thus, may rather represent a weak partial agonist, whereas 3HDC completely blocked TAS2R46 with IC_50_-concentrations in the low- to mid-micromolar range. Of note, 3HDC and 3HP, as well as to a lesser extent, GIV3727, also resulted in the activation of different subsets of TAS2Rs and therefore represent bitter compounds as well (Slack et al. 2010; Brockhoff et al. 2011). Perhaps the most striking example of the ambivalent nature of some bitter compounds/antagonists has been observed for the synthetic sweeteners saccharin and cyclamate (Behrens et al. 2017). Both sweeteners elicit a bitter off-taste at high concentrations through the activation of TAS2Rs; however, when blended, the bitter off-taste is reduced. It was demonstrated that both synthetic sweeteners exhibit mutual antagonistic activity by blocking, in the case of saccharin, the receptor for cyclamate (TAS2R1), and in the case of cyclamate, the saccharin-activated TAS2Rs (TAS2R31 and TAS2R43). Another natural bitter taste receptor antagonist, the plant hormone abscisic acid, has been identified for the TAS2R4 (Pydi et al. 2015). While the IC_50_-concentration of 4.5 µM indicates substantial potency, not all bitter substances activating TAS2R4 were blocked. Abscisic acid was not screened for the activation of all ∼25 functional human TAS2Rs; therefore, a wider range of antagonism and agonism cannot be excluded. Sakuranetin, a natural flavonoid, has been identified as a powerful antagonist for TAS2R31 (Fletcher et al. 2011). Another related group of compounds, the 6-methoxyflavanones, exhibited antagonism at the receptor TAS2R39 (Roland et al. 2014), which was further confirmed by the blocking of TAS2R39-mediated responses to the anti-HIV drug Tenofovir Alafenamide (Schwiebert et al. 2021). Interestingly, odorant molecules are also capable of blocking bitter taste receptors, as demonstrated by (R)-citronellal, which antagonizes TAS2R43 and TAS2R46 responses (Suess et al. 2018). A screening for antagonists against TAS2R8 resulted in the initial identification of a highly potent antagonist, called S5033 (Fotsing et al. 2020). Subsequent chemical modifications of S5033 led to two highly specific further TAS2R8 antagonists, S6821 and S7958. The blocking activity of S6821 against roasted coffee was confirmed by sensory experiments. While most of the above-mentioned antagonists may act as competitive inhibitors at the orthosteric binding site of TAS2Rs, allosteric antagonism of TAS2Rs was also found. The drug probenecid has been shown to bind to the intracellular regions of TAS2R16 and TAS2R38, leading to inhibition, possibly by interfering with signal transduction (Greene et al. 2011). Most recently, Fierro et al. successfully predicted in silico several antagonists (as well as novel agonists) of TAS2R14, indicating that computational methods may have reached accuracies capable of speeding up discoveries in this research direction (Fierro et al. 2023).

### TAS2R-variants

Soon after the discovery of human TAS2Rs, it was noticed that a considerable amount of genetic variation is present in the human population, suggesting that the bitter-tasting abilities of humans are quite individual (Wooding et al. 2004; Kim et al. 2005). To change the bitter perception of individuals, genetic variation must lead to changes in the receptors' functions, resulting in different phenotypes. The first bitter taste receptor gene with a proven functional polymorphism has been the TAS2R38 (Kim et al. 2003; Bufe et al. 2005). Historically, the discovery dates back to an observation by Fox, who studied the bitterness of phenylthiocarbamide (PTC) and found that the human population is split into individuals highly sensitive to this synthetic substance and individuals who are insensitive (Fox 1932). It took many decades, and, of course, the discovery of human TAS2R genes, before Kim and colleagues identified the responsible receptor, TAS2R38 (Kim et al. 2003). This receptor exists in two main variants in the human population, which are named after the amino acids underlying the functional differences, TAS2R38-PAV (the functional taster-variant) and TAS2R38-AVI (the nonfunctional nontaster variant) (Kim et al. 2003). Molecular evidence for the critical involvement of the variable positions, 49(P or A), 262 (A or V), and 296 (V or I), in the TAS2R38 polypeptide chain was provided by functional heterologous expression assays soon thereafter (Bufe et al. 2005). As the TAS2R38 not only responds to PTC or the later discovered 6-n-propyl-2-thiourea (PROP), but also to various additional compounds, such as goitrin (Wooding et al. 2010), methimazole (Behrens et al. 2013), and additional bitter substances in vegetables (Meyerhof et al. 2010), the TAS2R38 became the most studied of all TAS2Rs. Three main reasons promoted the rather rapid research success that pinpointed TAS2R38 as PTC-target and the identification of the crucial receptor positions: (i) the very high allele frequencies and hence their abundancies in the human population (about 30%–40% of the European and American Caucasians, about 5%–15% of the Japanese, and approximately 5% of the American Indian and Ainu populations are nontasters; Sato et al. 1997; Guo and Reed 2001; Wooding et al. 2004; Kim et al. 2005), (ii) the pronounced functional differences (functional versus nonfunctional, Bufe et al. 2005), and (iii) the unique specificity of this receptor for isothiocyanates and thioureas (Bufe et al. 2005). Although such beneficial circumstances were not found for any other receptor variant, several additional functional polymorphisms were reported. These include the TAS2R16 variant TAS2R16-K172N (Soranzo et al. 2005), the TAS2R9 variant TAS2R9-V187A (Dotson et al. 2008), the TAS2R31 variant TAS2R31-W35R (Pronin et al. 2007; Roudnitzky et al. 2011), and the TAS2R4 variant TAS2R4-SLN/FVS (Nolden et al. 2024). Additional associations of TAS2R polymorphisms with perceptual differences for bitter compounds were obtained by combinations of sensory experiments with genomic studies (for recent reviews, see Wooding et al. 2021; Wooding and Ramirez 2022). The total number of TAS2R variants within the genomes of humans suggests that a much larger number of functional variants could exist and are awaiting the discovery of unique, and of course, nontoxic bitter agonists for genetic linkage studies (Table 3). Of note are the rather large deviations of the fraction of nonsynonymous changes to the total number of variants, which fluctuates between 100% (TAS2R13) and 55.6% (TAS2R16) (Table 3, column %). This could indicate that the degree of evolutionary dynamics is different for different receptors. In fact, all TAS2Rs showing a fraction of 70% or below are either rather narrowly tuned or recognize distinct, and important, chemical classes of bitter compounds, which may require an elevated degree of conservation.

**Table 3.** Number of TAS2R variants found in the human population (data derived from Wooding and Ramirez 2022).

Receptor	# variants	Synonymous	Nonsynonymous	%
**TAS2R1**	27	6	21	77.8
**TAS2R3**	26	9	17	65.4
**TAS2R4**	29	7	22	75.9
**TAS2R5**	33	8	25	75.8
**TAS2R7**	40	9	31	77.5
**TAS2R8**	33	6	27	81.8
**TAS2R9**	31	4	27	87.1
**TAS2R10**	33	5	28	84.8
**TAS2R13**	15	0	15	100.0
**TAS2R14**	30	9	21	70.0
**TAS2R16**	36	16	20	55.6
**TAS2R19**	32	6	26	81.3
**TAS2R20**	37	9	28	75.7
**TAS2R30**	33	11	22	66.7
**TAS2R31**	40	4	36	90.0
**TAS2R38**	32	10	22	68.8
**TAS2R39**	35	9	26	74.3
**TAS2R40**	30	12	18	60.0
**TAS2R41**	32	13	19	59.4
**TAS2R42**	36	12	24	66.7
**TAS2R46**	33	7	26	78.8
**TAS2R50**	30	12	18	60.0
**TAS2R60**	35	11	24	68.6
**Sum**	738	183	543	74.6

“Nonsynonymous” including amino acid changes, stop-codons, insertions, deletions, etc. Pseudogenes and frequently deleted genes (TAS2R43 and TAS2R45) were excluded. % = percentage of nonsynonymous variants/total number of variants.

**Fig. 5.**
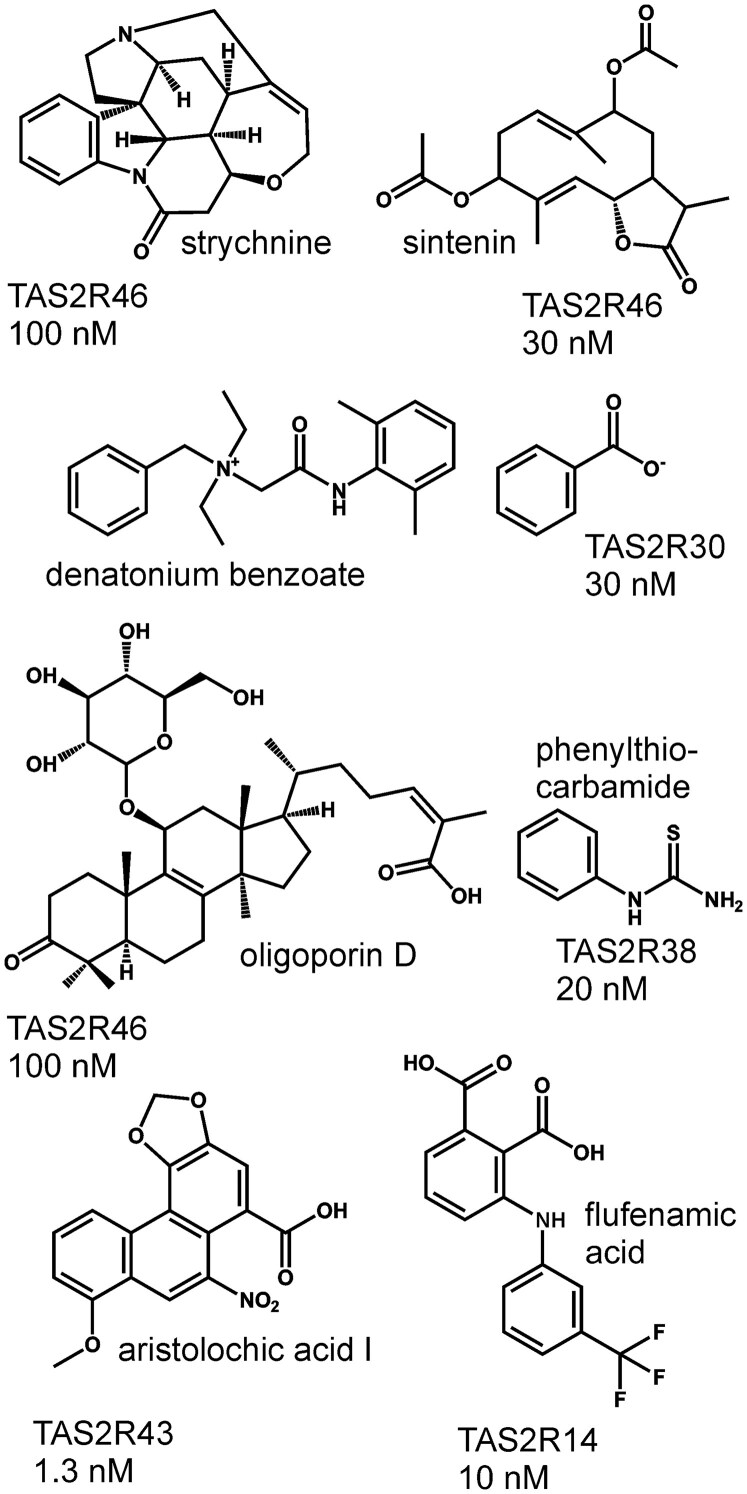
Exceptionally potent activators of TAS2Rs. Shown is a selection of some of the most potent activators together with the corresponding receptors and threshold concentrations (data taken from Kuhn et al. 2004; Pronin et al. 2004; Bufe et al. 2005; Brockhoff et al. 2007; Nowak et al. 2018; Schmitz et al. 2025).

In general, a very good agreement of the threshold concentrations obtained in sensory experiments and in vitro assays has been observed with only a few exceptions (for a recent review see Behrens 2025). Therefore, an important question in this regard is “what may be the reasons for a strong decorrelation between sensory data and in vitro assays”? One example for a much higher sensitivity of in vitro assays (c.f. Fig. 5 for a collection of extraordinarily potent bitter compounds) compared to sensory experiments is provided by the direct comparison of beer bitter compounds from hops (Intelmann et al. 2009). These experiments resulted in 20- to 1,000-fold higher concentrations necessary in sensory experiments compared to functional assays. One possible explanation that was already provided by the authors of this study could be perireceptor events, which reduce the effective compound concentration at the receptor site (Schmale et al. 1990; Spielman 1990; Matsuo 2000). Molecules known to interact with tastants in the oral cavity are, e.g. salivary proline-rich proteins (Bennick 1982). Some of these proline-rich proteins could interact with bitter compounds such as those from hops, thus preventing them from binding to TAS2Rs. Another possibility would be the absorption by mucins, another type of salivary proteins, or the epithelium of the oral cavity per se. Also in these cases, the effective concentration of tastants in the oral cavity could be lowered. Another rather receptor-specific observation concerns the reduction of TAS2R16 responses at low pH-values, which could occur in the oral cavity, but are not easily tolerated by mammalian cells used for functional assays (Sakurai et al. 2009). Moreover, enzymes, either secreted with saliva, associated with the rich microbiota present in the oral cavity, or located on oral epithelia or even the taste cells themselves (Chandrashekar et al. 2009; Sukumaran et al. 2016; Lossow et al. 2017), could modify taste stimuli.

Hence, there are numerous reasons for taste stimuli being apparently less potent in vivo than in vitro. However, what may be the reasons for those cases where the sensory detection is more sensitive than in vitro assays would suggest? One answer to this is that cultivated cells require a different environment compared to oral epithelia and may not tolerate bitter compound concentrations above a certain level. Whereas the sodium concentration in saliva is on average 28 mM (Adlin et al. 1969), mammalian cell lines in culture are typically maintained in ∼130 mM sodium chloride-containing buffers. It was shown recently that the in vitro response of the TAS2R16 to its prototypical agonist salicin (Bufe et al. 2002) is specifically reduced by about 39% (Kumar and Behrens 2024). In some instances, the cells used for functional assays may respond to high concentrations of a taste stimulus with responses independent of the presence of a TAS2R, and hence, a specific activation cannot be reported, although sensory experiments demonstrate a clear bitter perception. This leads to an apparent lack of sensitivity of the functional assay compared to sensory experiments, since in vivo bitterness is reported, whereas the assay outcome does not allow this conclusion. Such findings were, e.g. made with some bitter-tasting amino acids such as L-Arg, L-Ile, L-Leu, L-Tyr, and L-Val, which were reported to taste bitter, but could not be tested in vitro at sufficiently high concentrations (Kohl et al. 2013). Another possible explanation for compounds tasting bitter without activating a TAS2R in vitro is the existence of technical issues that precluded the deorphanization of one of the four yet orphan TAS2Rs, TAS2R19, -R42, -R45, and -R60 (Behrens 2025). One could assume that those compounds should target one of the orphan TAS2Rs. An example for such compounds would be naringin, which is reported to taste bitter (Guadagni et al. 1973), but in contrast to its aglycon naringenin, which activates TAS2R10 (Behrens et al. 2018) and/or TAS2R14/TAS2R39 (Roland et al. 2013), has not yet resulted in an association with one of the TAS2Rs. However, in the absence of clear hints on technical issues preventing the activation of the four orphan TAS2Rs in vitro and the bias of currently used bitter tastant libraries for synthetic and flowering plant-derived bitter compounds, such conclusions appear premature. There might be agonists for the orphan TAS2Rs in so far neglected sources for bitter compounds such as mushrooms (Schmitz et al. 2025).

### Structure-function relationships in TAS2Rs

**Fig. 6.**
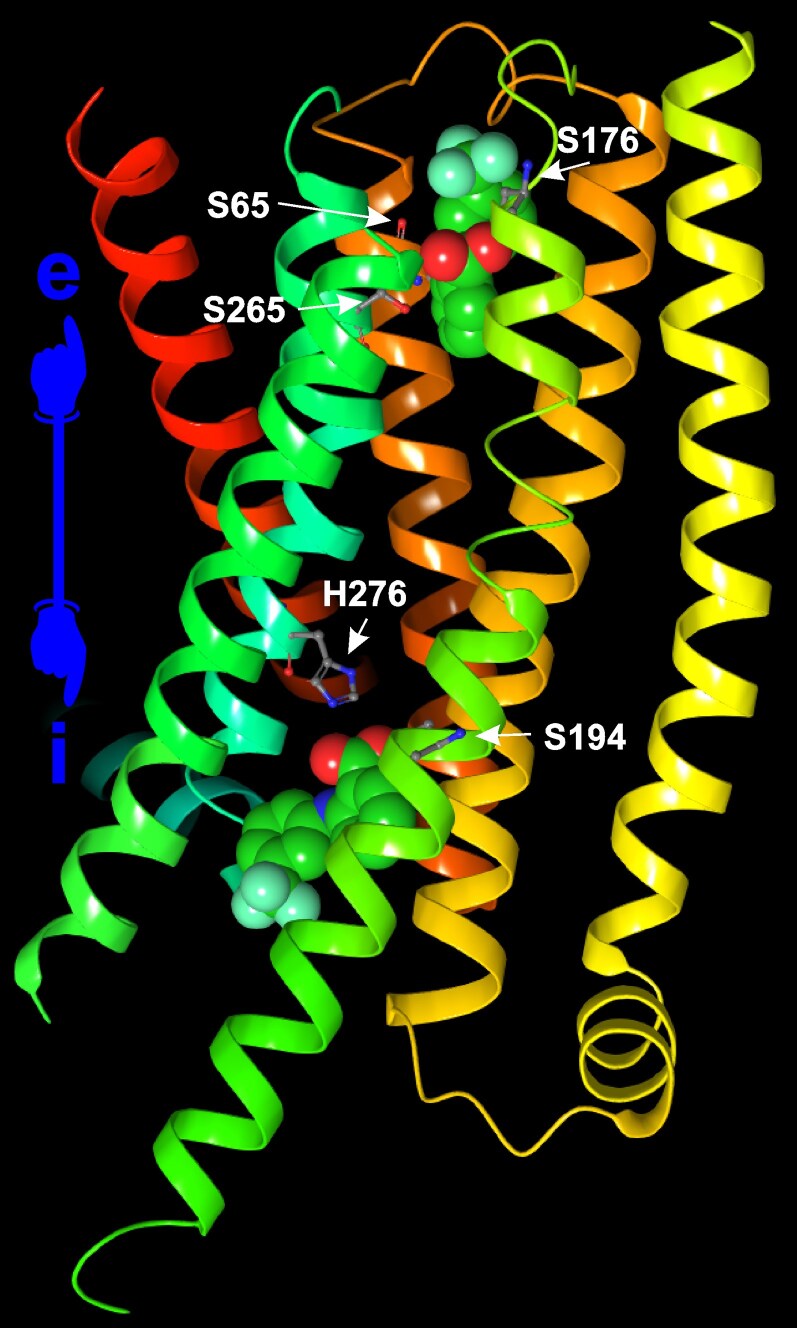
Three-dimensional representation of the TAS2R14 protein structure, showcasing its molecular configuration. The image highlights the protein's alpha-helical domains in ribbon style. The sign at the left indicates the orientation toward the extra- (e) and intracellular (i) side. The two flufenamic acid molecules are shown as space-filling models. The receptor residues forming hydrogen bonds with flufenamic acid are labelled and shown in ball-and-stick configuration. The illustration was generated from the PDB-file 8RQL (Peri et al. 2024) using Maestro 12.9 and CorelDRAW 2024.

Almost 20 years after the first researchers were brave enough to tackle the structures of the rather odd, low-homology G protein-coupled receptors of the TAS2R family by purely computational analyses (Floriano et al. 2006; Miguet et al. 2006), a recent series of experimental cryo-EM structures has added to our knowledge about human TAS2Rs. Among the five recently published experimental structures, four were devoted to the most broadly tuned receptor, the TAS2R14 (Hu et al. 2024; Kim et al. 2024 ; Peri et al. 2024; Tao et al. 2024), whereas one study was done on TAS2R46 (Xu et al. 2022), and the most recent publication targeted TAS2R16 (Wang et al. 2025) (Table 4, Fig. 6). As the four experimental studies on TAS2R14 showed substantial deviations in the interpretation of the experimental results, previously published studies arising from combined efforts of computational and experimental research should be taken into account.

**Table 4.** Comparison of the binding sites described for the recently determined cryo-EM structures of TAS2Rs.

	Receptor					
	TAS2R14				TAS2R16	TAS2R46
	Hu et al.	Kim et al.	Peri et al.	Tao et al.	Wang et al.	Xu et al.
**TM2 I62^2.57^** **TM2 S63^2.57^** **TM2 L62^2.57^**		Cholesterol (orthosteric)		28.1 (orthosteric)	Salicin	Strychnine
**TM2 S65^2.60^** **TM2 N66^2.60^** **TM2 N65^2.60^**			Flufenamic acid (extracellular)	Cholesterol (orthosteric) 28.1 (orthosteric)	Salicin	Strychnine
**TM2 N67^2.61^**					Salicin	
**ECL1 F76**	Cholesterol (pocket 1)	Cholesterol (orthosteric)	Flufenamic acid (extracellular)	Cholesterol (orthosteric) 28.1 (orthosteric)		
**TM3 F82^3.25^**	Cholesterol (pocket 1)	Cholesterol (orthosteric)	Flufenamic acid (extracellular)	Cholesterol (orthosteric) 28.1 (orthosteric)		
**TM3 L85^3.28^**		Cholesterol (orthosteric)				
**TM3 T86^3.29^** **TM3 Y85^3.29^**			Flufenamic acid (extracellular)	Cholesterol (orthosteric) 28.1 (orthosteric)		Strychnine
**TM3 W89^3.32^** **TM3 W85^3.32^** **TM3 W88^3.32^**	Cholesterol (pocket 1)	Cholesterol (orthosteric)	Flufenamic acid (extracellular)	Cholesterol (orthosteric) 28.1 (orthosteric)	Salicin	Strychnine
**TM3 Y107^3.50^**	Aristolochic acid (pocket 2) Flufenamic acid (pocket 2) 28.1 (pocket 2)	28.1 (allosteric)	Flufenamic acid (intracellular)	28.1 (allosteric)		
**TM3 I111^3.54^**	Aristolochic acid (pocket 3)	28.1 (allosteric)	Flufenamic acid (intracellular)			
**ECL2 F172**			Flufenamic acid (extracellular)			
**ECL2 R174**				Cholesterol (orthosteric)		
**ECL2 S176**			Flufenamic acid (extracellular)	28.1 (orthosteric)		
**TM3 I179^5.40^**		Cholesterol (orthosteric)				
**TM3 V180^5.41^**		Cholesterol (orthosteric)		Cholesterol (orthosteric) 28.1 (orthosteric)		
**TM3 T180^5.43^**						Strychnine
**TM3 T184^5.45^**		Cholesterol (orthosteric)		Cholesterol (orthosteric)		
**TM3 T187^5.47^**		Cholesterol (orthosteric)		Cholesterol (orthosteric)		
**TM5 F188^5.48^**		Cholesterol (orthosteric)				
**TM5 S194^5.54^**	Aristolochic acid (pocket 2) Flufenamic acid (pocket 2) 28.1 (pocket 2)	28.1 (allosteric)	Flufenamic acid (intracellular)	28.1 (allosteric)		
**TM5 F198^5.58^**	Aristolochic acid (pocket 2) Flufenamic acid (pocket 2) 28.1 (pocket 2)	28.1 (allosteric)	Flufenamic acid (intracellular)	28.1 (allosteric)		
**TM5 L201^5.61^**	Flufenamic acid (pocket 2) 28.1 (pocket 2)	28.1 (allosteric)	Flufenamic acid (intracellular)	28.1 (allosteric)		
**TM5 L202^5.62^**			Flufenamic acid (intracellular)			
**TM5 H208^5.68^**	Aristolochic acid (pocket 3)					
**TM5 R209^5.69^**	Aristolochic acid (pocket 3)					
**TM6 S223^6.28^**	Aristolochic acid (pocket 3)					
**TM6 H227^6.32^**	Aristolochic acid (pocket 3)					
**TM6 V230^6.35^**	Aristolochic acid (pocket 3) Flufenamic acid (pocket 2) 28.1 (pocket 2)		Flufenamic acid (intracellular)			
**TM6 V233^6.38^**	Aristolochic acid (pocket 2) Flufenamic acid (pocket 2) 28.1 (pocket 2)	28.1 (allosteric)	Flufenamic acid (intracellular)	28.1 (allosteric)		
**ECL3 F252**						Strychnine
**TM6 F237^6.42^**		28.1 (orthosteric)	Flufenamic acid (intracellular)			
**TM6 Y240^6.45^**				28.1 (allosteric)		
**TM6 F247^6.52^**		Cholesterol (orthosteric)		Cholesterol (orthosteric)		
**TM7 I262^7.34^**		Cholesterol (orthosteric)	Flufenamic acid (extracellular)	Cholesterol (orthosteric)		
**TM7 S265^7.38^**			Flufenamic acid (extracellular)	28.1 (orthosteric)		
**TM7 Q266^7.39^** **TM7 E262^7.39^** **TM7 E265^7.39^**	Cholesterol (pocket 1)		Flufenamic acid (extracellular)	Cholesterol (orthosteric) 28.1 (orthosteric)	Salicin	Strychnine
**TM7 M268^7.41^**				Cholesterol (orthosteric)		
**TM7 Y272^7.45^**	Aristolochic acid (pocket 2)					
**TM7 H276^7.49^**	Aristolochic acid (pocket 2) Flufenamic acid (pocket 2) 28.1 (pocket 2)	28.1 (allosteric)	Flufenamic acid (intracellular)	28.1 (allosteric)		
**TM7 G283^7.56^**			Flufenamic acid (intracellular)			

The involved receptor positions for TAS2R14, TAS2R16, and TAS2R46 are provided (code = domain (TM = transmembrane domain, ECL = extracellular loop); amino acid position^Ballesteros–Weinstein–Nomenclatur^). If more than a single binding pocket was proposed, the compound name (compound 28.1 = 28.1) is given together with the specified binding pocket (pocket 1 to 3, orthosteric binding pocket (orthosteric), extracellular binding pocket (extracellular), allosteric binding pocket (allosteric), intracellular binding pocket (intracellular).

All four structural studies on the TAS2R14 agree about the existence of at least one additional binding pocket that is oriented toward the intracellular side of the receptor. This raises the imminent question about the accessibility of compounds to reach this site. In general, three scenarios could be envisaged. Firstly, the ligand enters from the extracellular side, passing the orthosteric binding site to reach the intracellularly oriented binding site. According to Kim et al., the TAS2R14 structure exhibits a tunnel-like space connecting the orthosteric with the intracellular binding site, which could allow entry of ligands from extra- and intracellular sides (Kim et al. 2024). Moreover, secondly, tunnels that would allow entry of ligands from the membrane compartment were proposed as well (Kim et al. 2024 ). Thirdly, in contrast to such a passageway through the receptor, Peri and colleagues discussed the possibility that the intracellular site predominantly interacts with membrane-permeable or intracellular ligands (Peri et al. 2024). Regardless of the different scenarios by which ligands may target the different binding sites in TAS2R14, all of them would add features necessary to qualify bitter compounds for TAS2R14 interaction. A compound that enters the TAS2R14 from the extracellular side and crawls through a tunnel to reach the intracellular binding site should avoid high-affinity contacts while passing the orthosteric site and tunnel but then afford binding to the intracellular pocket. Similarly, a ligand that needs to traverse the membrane first to reach the intracellular pocket should either be amphiphatic for diffusion or be actively transported into the cell. This suggests that intracellular ligands of TAS2R14 might be less diverse compared to the extracellular ones.

Also, regarding the TAS2R14 positions involved in agonist binding, some differences are evident. While all reports on TAS2R14 agree with the absence of ligand-receptor interactions in TM1 and TM4, Hu et al. additionally observed a lack of TM2 involvement; hence, all reports agree that positions in TM3, TM5, TM6, and TM7 facilitate ligand contacts. Although the studies on TAS2R14 involved a variety of different agonists, several TAS2R14 positions have been implicated in agonist interactions throughout. These were F76 in ECL1, in TM3 positions F82^3.25^, W89^3.32^, Y107^3.50^; in TM5 S194^5.54^, F198^5.58^, L201^5.61^; in TM6 V233^6.38^; in TM7 H276^7.49^. Numerous additional positions are shared among two or three studies. However, eight positions are only implicated by one of the four studies, indicating that experimental data also exhibit some variability. Interestingly, the two experimental studies on receptors other than TAS2R14 show fewer points of interaction between TAS2R16 and salicin and TAS2R46 and strychnine, respectively. One of the reasons for this is, of course, the fact that these receptors possess only a single orthosteric agonist binding site; however, it also indicates that TAS2R16 and TAS2R46 are not as broadly tuned as TAS2R14.

## Other vertebrates' bitter taste receptors

**Fig. 7.**
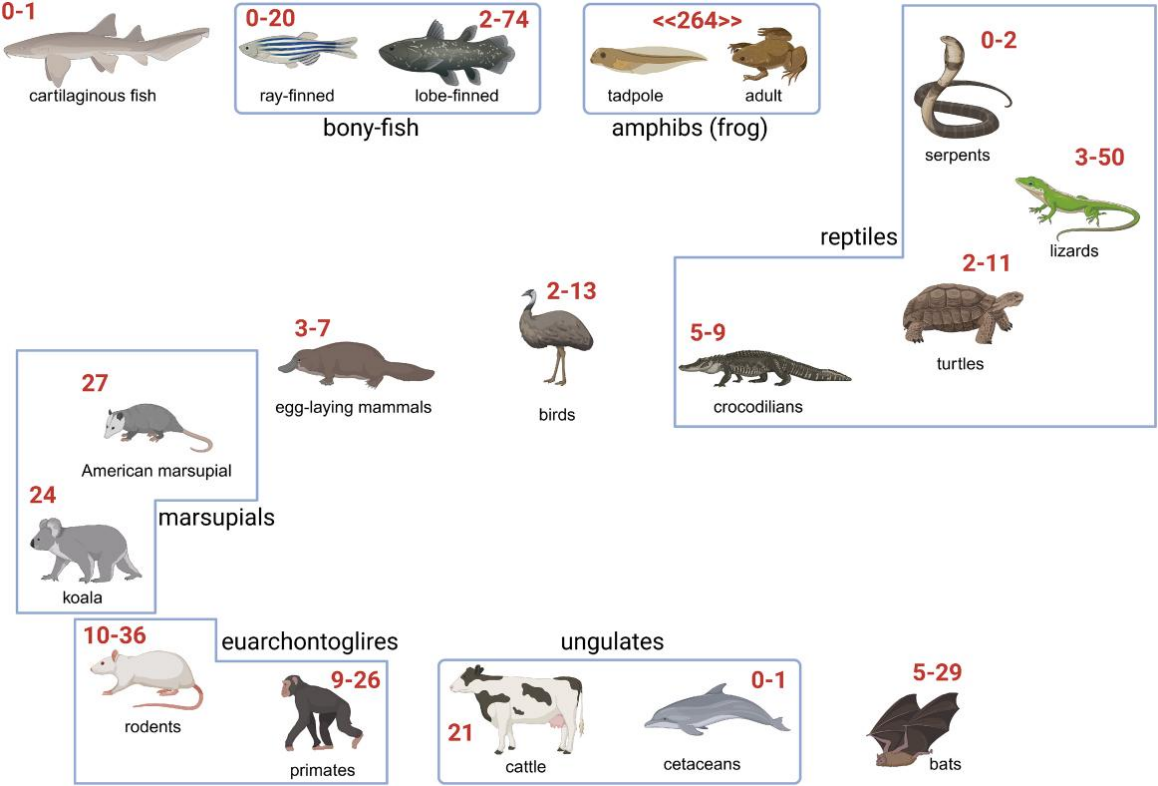
Evolution of T2R gene repertoires. Shown is a selection of species for which the number of putatively functional bitter taste receptor genes has been determined. Created in BioRender. Behrens, M. (2025) https://BioRender.com/7476xtd.

While research on vertebrate bitter taste receptors in the early years focused exclusively on human and rodent receptors, data on numerous additional species are available now and keep emerging at a rapid pace. To date, we have information about the bitter taste receptor gene repertoires of at least one, and mostly several, representatives of the majority of jawed vertebrate (Gnathostomata) clades (Fig. 7).

The rapid increase in knowledge about vertebrate bitter taste receptor genes is mostly driven by the expanded availability of genomic data; however, the amount of functional data also steeply increases. In two recent, very comprehensive publications, the bitter taste receptor repertoires of 1,527 and 661 vertebrate genomes were analyzed, respectively (Policarpo et al. 2024; Higgins et al. 2025). The analyzed genomes ranged from lampreys (agnatha) over cartilaginous fish, ray-finned fish, lungfish, amphibians, reptiles, and birds, to mammals. The number of putatively intact bitter taste receptor genes ranged from 0 in jawless species to 248 (wood frog; Higgins et al. 2025) and 264 (Japanese wrinkled frog; Policarpo et al. 2024) *T2Rs* in amphibian genomes (unlike for human and mouse bitter taste receptors, gene symbols for other vertebrates have not been fixed by nomenclature commissions, therefore we use here the symbol “T2R/*T2R*”). The documented evolutionary first appearance of a single *T2R* gene occurred in cartilaginous fish (Behrens et al. 2023; Itoigawa et al. 2024). From there, the number of *T2R* genes mostly increased with considerable differences in bony fish. Putative functional T2Rs in ray-finned fish (Actinopterygii) range from 0 in the electric knife fish (*Electrophorus electricus*) to 20 in Mexican cave-fish (*Astyanax mexicanus*), with the majority of fish in the one-digit scale (Behrens et al. 2021). In the lobe-finned lineage (Sarcopterygii), the West African lungfish (2 intact T2Rs; Behrens et al. 2023 in *Protopterus annectens*) possesses a much smaller *T2R* gene repertoire compared to the West Indian Ocean Coelacanth (74; Behrens et al. 2021 in *Latimeria Chalumnae*). Among the amphibians, there is a pronounced difference in the number of T2R, with cecilians possessing a rather small number and batrachians showing the highest average T2R number of all vertebrates (Higgins et al. 2025). An attractive idea for the development of elevated *T2R* gene numbers in some batrachian species is the substantial change in habitats before and after metamorphosis. Indeed, Hao and colleagues reported the differential expression of multiple *T2R* genes in tadpoles and adults of the American Bullfrog. Whereas the aquatic and herbivorous tadpole show enrichment of fewer and less broadly tuned T2Rs, the terrestrial and insectivorous adults express more, and more broadly tuned T2Rs. This indicates that a dietary shift during ontogenesis may require the activities of more functionally different specialized T2Rs (Hao et al. 2023). The number of intact *T2R* genes in reptiles fluctuates considerably. Whereas most snakes possess only between 0 (common garter snake) and 2 T2Rs, lizard genomes typically exhibit 3 to 50 *T2R* genes (Zhong et al. 2017, 2019). The turtles carry T2R repertoires with rather low to medium numbers, typically between 2 and 11 (Zhong et al. 2017; Behrens et al. 2021). The crocodilians, which are most closely related to birds, tend to possess only a few *T2R* genes (5 to 9) (Zhong et al. 2017). Similarly, birds with 2 (emu, turkey) to 13 (northern cardinal) show *T2R* gene numbers at the lower end of the scale (Behrens et al. 2014). Concerning mammals, egg-laying mammals, a sister clade of marsupials and placentals, possess only 3 (echidna) to 7 (platypus) *T2R* genes (Zhou et al. 2021; Itoigawa et al. 2022). Like placentals (eutherians), marsupials exhibit considerably more putatively functional *T2R* genes. With 24 T2Rs, the koala has the most functional T2Rs of all Australian marsupials (Johnson et al. 2018), whereas the opossum, a marsupial living on the American continent, has a slightly higher T2R repertoire (27 *T2R* genes) (Li and Zhang 2014). Also, among the higher mammals, the numbers of potentially intact T2R genes fluctuate considerably. The glires clade, including the rodents, possesses between 10 and 36 T2Rs, and similarly, the euarchonta, including the primates, 9 to 26 (Li and Zhang 2014). Whereas ungulates carry up to 21 T2Rs (Bos Taurus), cetaceans, which conquered aquatic habitats, typically do not possess *T2R* genes (Li and Zhang 2014). Next to rodents, bats are the second largest group of mammals. While they typically contain 15 to 29 *T2R* genes in their genomes (Li and Zhang 2014), the exclusively blood-feeding vampire bats have only 5 to 6, with 3 orthologous genes present in all 3 species (Ziegler and Behrens 2021).

On a functional level, most vertebrate T2Rs strongly align with features originally described for human TAS2Rs (for a recent review, see Behrens 2025). They can deviate in their tuning breadths from narrow to broad (Behrens et al. 2014), the threshold concentrations for activation lie almost always in the micromolar range, and they can interact with antagonistic molecules (Behrens et al. 2021), although much fewer antagonists against nonhuman T2Rs are reported at present. While it was observed previously that functional conservation between human and mouse bitter taste receptors is scarce (Lossow et al. 2016), evidence for an astonishing level of functional conservation between even more distantly related species is accumulating. Examples for the latter are the full conservation of agonist profiles among the single cartilaginous fish T2R1 in bamboo and catshark (Behrens et al. 2023), the similarly high conservation among coelacanth lcT2R01 and zebrafish zfT2R1 (Behrens et al. 2021), or the excellent conservation of metal ion responsiveness among human TAS2R7 and *Desmodus rotundus* (vampire bat) T2R7 (Ziegler and Behrens 2021). Despite separation times between ≥90 and ≥400 Mya, the agonist activation patterns did not change, indicating strict functional conservation, which may not apply to the individual agonists.

Quite a few publications have been devoted to correlating the number of T2R genes a species carries with dietary habits. While several studies observed that herbivores possess more putatively functional *T2R* genes compared to carnivores (e.g. Li and Zhang 2014), other studies found that omnivores have more functional T2Rs compared to herbivores (Policarpo et al. 2024). It seems that good agreement exists for the fact that carnivores rarely encounter potentially poisonous bitter compounds in feed stuff and hence, rely less on their bitter taste receptor repertoires for survival. However, a recent study on folivorous and omnivorous primates indicated that gain and loss of *T2R* genes occurred at almost every phylogenetic tree branch, which suggests that the evolutionary dynamics of bitter taste receptors may be more species-specific than reflected by generalized dietary habits (Hou et al. 2024).

## A brief excursion into extraoral bitter taste receptors

### Tissue distribution

**Fig. 8.**
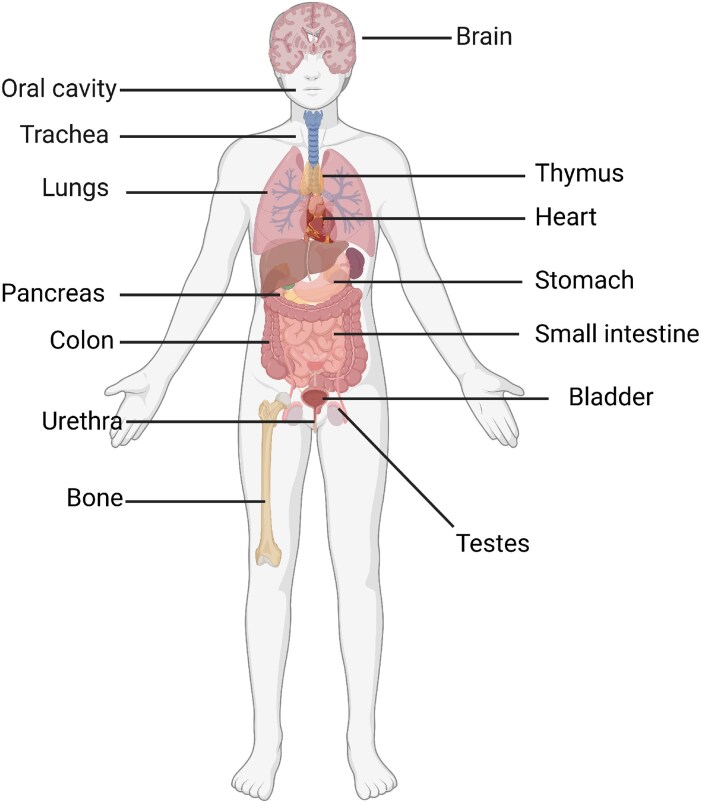
Extra-oral expression of taste GPCRs, sensing bitter, sweet, and umami compounds. Originally discovered in the oral cavity, taste receptors are distributed throughout the body. There, they trigger important physiological roles, which have yet to be revealed completely. Modified from Lee and Cohen (2016) and excluding the likewise identified taste receptor-expressing blood cells. Created in BioRender. Schäfer, S. (2025) https://BioRender.com/oia0xrq.

Various chemosensory epithelial cells outside the gustatory system express taste-specific features and are scattered throughout numerous organs and tissues of mammalian species (Finger and Kinnamon 2011). In fact, canonical taste-signaling components such as α-gustducin, PLCβ2, or TRPM5 were found in tissues other than the oral cavity, such as in the airways and the gastrointestinal tract (Höfer et al. 1996; Finger et al. 2003; Bezençon et al. 2007; Lin et al. 2008). Additionally, the extensive distribution of taste GPCRs throughout the body proposes functional roles beyond taste sensation (for recent comprehensive reviews see Wang et al. 2020; D'Urso and Drago 2021; Tuzim and Korolczuk 2021; Behrens and Lang 2022). An overview of tissues expressing taste GPCRs is given in Fig. 8.

### Physiological roles

Some effects of activated extraoral taste receptors were observed in the GI tract, except for the testis (Matsunami et al. 2000), the first tissue for which taste receptor expression outside the oral cavity was identified (Wu et al. 2002). Enteroendocrine cells express taste GPCRs and are involved in sensing metabolites and food components, assisting in hormone regulation such as glucagon-like peptide 1 (GLP-1) and ghrelin (for a review, see Behrens and Meyerhof 2019). Both TAS1R heteromers are located on secretory stomach cells. When excited by umami or sweet tastants, they release the “hunger” hormone ghrelin, thus increasing food intake (Hass et al. 2010; Finger and Kinnamon 2011). Sweet taste receptors are further located on duodenal L-cells. Upon stimulation, these cells release GLP-1 (Jang et al. 2007), increasing insulin secretion. Additionally, TAS1R2/TAS1R3 heterodimers are located on the β-cell surface, presenting a second pathway for insulin secretion (Henquin 2012; Kyriazis et al. 2012). Further, the insertion of other glucose transporters in the apical intestinal cell membrane is increased upon sweet taste receptor activation (Mace et al. 2007; Margolskee et al. 2007). This modulation in GLP-1 release may offer a significant treatment option for diabetes or obesity (Jang et al. 2007). In addition, representatives of the TAS2R family have been discovered in the GI tract. Thus, the activation of a bitter taste receptor expressed in enteroendocrine cells leads to the secretion of GLP-1 (Dotson et al. 2008) followed by the previously mentioned effects. Moreover, the activation of TAS2Rs causes the secretion of cholecystokinin (CCK), which diminishes stomach emptying and transmits satiety. This fact was previously proven by the discovery of denatonium benzoate having the predicted effects (Glendinning et al. 2008; Hao et al. 2008). When TAS2Rs are activated in the colonic area of the GI tract, the secretion of anions into the gut lumen is induced. This provokes fluid excretion from the epithelium, potentially for the clearance of harmful compounds (Kaji et al. 2009).

Zancanaro and colleagues were the first to find taste-linked α-gustducin-expressing cells present in the vomeronasal organ (Zancanaro et al. 1999). The morphology of these cells had already been revealed before in fish and has since been designated as solitary chemosensory cells (SCCs) (Finger 1997). These SCCs were then found to be spread all over the airways of rodents and humans, including further taste-related signaling components (Finger et al. 2003; Kaske et al. 2007). It was shown that these chemosensory cells need the taste-signaling cascade for activation (Finger et al. 2003). The signals of the activated SCCs modulate trigeminally mediated reflex alterations in respiration (Finger and Kinnamon 2011) and are therefore taste perception independent. Just as the GI tract, the respiratory system is directly exposed to the exterior environment. Thus, these systems are in contact with a wide variation of bacteria, viruses, and fungi, including their metabolites. For this reason, it poses a challenge for these tissues to avoid infections. Experimental data suggest a role of bitter taste receptors in signaling and mediating innate immunity in the epithelia. TAS2Rs are expressed on ciliated epithelial cells in human and rodent airways (Lu et al. 2017). Upon the sensing of bitter compounds, the previously explained taste GPCR signaling pathway is induced, leading to the rise of intracellular Ca^2+^ concentration, implementing an increase of the ciliary beat frequency, augmenting the clearance of microorganisms and originating products (Shah et al. 2009). Further, the TAS2R38 is another example of the participation of bitter receptors in the innate immune response. This GPCR is expressed in the apical membrane and cilia of the sinus epithelium. Besides known agonists, quorum-sensing molecules have been proposed to activate this receptor (Lee et al. 2012, but c.f. Verbeurgt et al. 2017). When the concentration of quorum-sensing molecules is sufficiently high, a biofilm is created that protects the bacteria from the immune defense system of the host organism (Marx 2014). Upon activation of TAS2R38, nitric oxide (NO), a very effective bactericide, is generated (Lee et al. 2012; Lee and Cohen 2013). Additionally, NO increases the ciliary beat frequency (Shaik et al. 2016), and, as a consequence, increases the clearing rate. This effect depends on the canonical taste signaling components TRPM5 and PLCβ2 (Lee and Cohen 2014). Moreover, the mentioned antimicrobial effects of TAS2Rs are controlled by TAS1R2/TAS1R3. Thus, the sweet taste receptor diminishes the bitter GPCR activation and therefore the antimicrobial peptide secretion, when the health status is relatively high (Lee and Cohen 2014; Lee et al. 2014). Other functional roles of TAS2Rs can be found in the lower airways, e.g. in asthma. Traditionally, asthma is treated with anti-obstructive drugs, such as β2-adrenergic receptor agonists or anti-inflammatory corticosteroids (Holgate et al. 2015). Bitter receptors may turn out to be important targets in therapeutic treatment. Functional bitter receptors were found in human airway smooth muscle cells. Upon stimulation with bitter compounds, these GPCRs cause the relaxation of the smooth muscle by increasing Ca^2+^ levels and subsequently hyperpolarization of the smooth muscle cells. In an animal model, it was shown that TAS2Rs mediate this relaxation in a dose-dependent manner, and therefore, bitter compounds may represent a treatment strategy in addition to β2-adrenergic receptor agonists (Deshpande et al. 2010).

In addition to tissues directly in contact with the exterior environment, taste receptors are also expressed in internal tissues, such as the thyroid gland. The thyroid hormones thyroxine and triiodothyronine are important for thriving (Gereben et al. 2008). An interesting study revealed the expression of TAS2Rs in thyrocytes as well as an involvement of bitter receptors in thyroid hormone synthesis (Clark et al. 2015). Moreover, it was found that upon TAS2R activation, the efficacy of thyroid-stimulating hormone (TSH) is diminished. Hence, the TSH-dependent iodide efflux from thyrocytes is modified and thus the secretion of thyroxine and triiodothyronine. Moreover, TAS2R polymorphisms in thyrocytes have been shown to modify circulating levels of thyroid hormones (Clark et al. 2015).

It is also hypothesized that taste receptors may function as nutrient sensors in the heart. Foster *et al.* found expression of bitter and umami taste receptors in human and rodent heart tissue (Foster et al. 2013). Taste signaling compounds such as *Plcβ2* and *Trpm5* are expressed in cardiomyocytes as well. Specifically, Tas1r1/Tas1r3 heterodimers were found in myocardial tissue, implementing an amino acid-sensing and possible metabolic regulatory role of this GPCR. Previously, this heterodimer was identified as a direct upstream amino acid sensor for an autophagy pathway (Wauson et al. 2012). Regarding TAS2Rs, an upregulation upon nutrient deprivation was observed, indicating a role as a sensor in cardiac tissue (Foster et al. 2013), which is further substantiated by the impact of bitter compounds on heart function (Foster et al. 2014).

### Possible endogenous agonists

Recently, it was found that inorganic molecules such as di- and trivalent metal salts activate the human TAS2R7 and its vampire bat ortholog drTas2r7 (Behrens et al. 2019; Wang et al. 2019; Ziegler and Behrens 2021). Although ultimately ingested as part of food and beverages, metal ions play crucial roles in endogenous processes. Magnesium, for instance, is clinically used for the treatment of conditions such as preeclampsia, stroke, myocardial infarction, or bowel-associated diseases, such as diarrhea, colitis, or gastritis (Feldman 1999; de Baaij et al. 2015). Iron is required by humans for oxygen transport and utilization, cellular proliferation, energy production, and pathogen obliteration (Lynch et al. 2018). Transcription factors and enzymes containing copper are fundamental for cellular signaling, integrity, proliferation, energy production, radiation defense, and oxidation (Kardos et al. 2018). Manganese is a cofactor for several enzymes; it is essential for the normal function of several physiologic processes, including protein glycosylation and gluconeogenesis (Avila et al. 2013). Zinc plays a crucial role in endogenous processes such as signal transduction (Krzywoszyńska et al. 2020). Thus, metal ions may be candidates for endogenous agonists.

As a further, and this time bona fide putative endogenous agonist, bile acids were identified. Interestingly, the threshold concentrations found to elicit responses in TAS2Rs matched concentrations of bile acids found in human body fluids. This fact underscores the possibility for the activation of extraoral bitter taste receptors by endogenous agonists (Ziegler et al. 2023). Furthermore, it was found that the activation of bitter taste receptors by a variety of bile acids is also conserved between distantly related species, such as human, mouse, frog, and chicken (Schaefer et al. 2024) thus indicating evolutionary conservation.

## Summary

In the last 25 years, after the initial discovery of bitter taste receptors, enormous progress has been made. The majority of TAS2Rs have been deorphaned, and numerous bitter taste receptor gene repertoires of other vertebrates were analyzed genomically and partly functionally, thus providing a basis for comparisons. So far, nothing hints at particular “human” features of our sense of bitter taste in general (sensitivity differences for select compounds excluded). After deorphanization and the identification of increasingly more bitter agonists per TAS2R, the discovery of receptor-specific bitter inhibitors represented a major advancement, now allowing us to use them as novel research tools as well as for applications in drug development, an area with great potential. While structure-function studies of bitter taste receptors and their ligands were, in the past, very laborious and hampered by the low degree of homology with experimental structures of other GPCRs, the current burst of newly published cryo-EM structures may lead to increased accuracy and a speed-up of discoveries related to TAS2R-ligand interactions. Also, the field of nongustatory bitter taste receptor research has seen enormous progress. While extra-oral bitter taste receptors seemed to be limited initially to the gastrointestinal tract (Wu et al. 2002) and hence indicated an “elongated” route of nutrient/xenobiotics-sensing, nowadays most tissues are implicated in bitter taste receptor agonist-sensing. There are even emerging hints that some agonists for bitter taste receptors represent endogenous molecules. Despite this enormous progress, there are many open questions newly emerging or simply persisting. What might be the reason for the remaining orphan TAS2Rs to resist deorphanization? Are these technical issues, or do we simply not have the right activators in hand, because our knowledge about our bitter surroundings is still too shallow? Another issue is the mostly rather loose association of bitter taste receptors with the implicated nongustatory functions. Some carefully performed recent studies have raised concerns about the involvement of bitter taste receptors in processes frequently assumed to be firmly established (Perniss et al. 2020; Lu et al. 2021). Here, the development of highly specific antibodies would certainly provide wonderful tools to tighten the links between receptors and the observed nongustatory processes. Finally, the findings of bitter taste receptors in gustatory and nongustatory tissues, as well as the hints for endogenously synthesized bitter agonists, raises the question of the original function of these intriguing molecules.

## Data Availability

No new data were generated or analyzed in support of this research.
